# MicroRNA-21 is a candidate driver gene for 17q23-25 amplification in ovarian clear cell carcinoma

**DOI:** 10.1186/1471-2407-14-799

**Published:** 2014-11-03

**Authors:** Yukihiro Hirata, Noriyuki Murai, Nozomu Yanaihara, Misato Saito, Motoaki Saito, Mitsuyoshi Urashima, Yasuko Murakami, Senya Matsufuji, Aikou Okamoto

**Affiliations:** Department of Obstetrics and Gynecology, The Jikei University School of Medicine, 3-25-8, Nishi-Shinbashi, Minato-ku, Tokyo, 105-8461 Japan; Department of Molecular Biology, The Jikei University School of Medicine, 3-25-8, Nishi-Shinbashi, Minato-ku, Tokyo, 105-8461 Japan; Division of Molecular Epidemiology, The Jikei University School of Medicine, 3-25-8, Nishi-Shinbashi, Minato-ku, Tokyo, 105-8461 Japan

**Keywords:** Ovarian clear cell carcinoma, CGH array, microRNA-21, PTEN

## Abstract

**Background:**

Epithelial ovarian cancer (EOC) is the most common cause of gynecological malignancy-related mortality. Ovarian clear cell carcinoma (CCC) has unique clinical characteristics and behaviors that differ from other histological types of EOC, including a frequent association with endometriosis and a highly chemoresistant nature, resulting in poor prognosis. However, factors underlying its malignant behavior are still poorly understood. Aberrant expression of microRNAs has been shown to be involved in oncogenesis, and *microRNA-21* (*miR-21*) is frequently overexpressed in many types of cancers. The aim of this study was to investigate the role of *miR-21* in 17q23-25 amplification associated with CCC oncogenesis.

**Methods:**

We identified 17q23-25 copy number aberrations among 28 primary CCC tumors by using a comparative genomic hybridization method. Next, we measured expression levels of the candidate target genes, *miR-21* and *PPM1D*, for 17q23-25 amplification by real-time RT-PCR analysis and compared those data with copy number status and clinicopathological features. In addition, immunohistochemical analysis of PTEN (a potential target of *miR-21*) was performed using the same primary CCC cases. We investigated the biological significance of *miR-21* overexpression in CCC using a loss-of-function antisense approach.

**Results:**

17q23-25 amplification with both *miR-21* overexpression and PTEN protein loss was detected in 4/28 CCC cases (14.2%). The patients with 17q23-25 amplification had significantly shorter progression-free and overall survival than those without 17q23-25 amplification (log-rank test: p = 0.0496; p = 0.0469, respectively). A significant correlation was observed between *miR-21* overexpression and endometriosis. Both *PTEN* mRNA and PTEN protein expression were increased by *miR-21* knockdown in CCC cells. We also confirmed that *miR-21* directly bound to the 3′-untranslated region of *PTEN* mRNA using a dual-luciferase reporter assay.

**Conclusions:**

*MiR-21* is a possible driver gene other than *PPM1D* for 17q23-25 amplification in CCC. Aberrant expression of *miR-21* by chromosomal amplification might play an important role in CCC carcinogenesis through the regulation of the *PTEN* tumor suppressor gene.

**Electronic supplementary material:**

The online version of this article (doi:10.1186/1471-2407-14-799) contains supplementary material, which is available to authorized users.

## Background

Epithelial ovarian cancer (EOC), a heterogeneous group of neoplastic diseases that arise from the epithelial cells of fallopian tubes, ovarian fimbria, ovarian surface epithelium, inclusion cysts, peritoneal mesothelium, or endometriosis, is the most lethal gynecologic malignancy in western countries and in Japan [1]. EOC can be classified into four major histological types: serous, mucinous, endometrioid adenocarcinoma, and clear cell carcinoma (CCC). CCC has unique clinical characteristics that differ from other histological types of EOC. CCC accounts for 5–25% of all EOC, depending on the population. The prevalence of CCC among EOCs in North America and Europe is 1–12%, while that in Japan is approximately 20% [2]. CCC is frequently associated with coexistent endometriosis and thrombosis, with 20% of patients developing deep venous thrombosis. Endometriosis has been identified in more than 30% of tumors and is reported to be a precursor of CCC as well as endometrioid adenocarcinoma [3]. The incidence of venous thromboembolic events was found to be significantly higher in CCC than in other epithelial ovarian cancers [4, 5]. A greater proportion of CCC presents in the early stage as a large pelvic mass, which may account for their earlier diagnosis. However, CCC is generally refractory to standard platinum agent-based chemotherapy with a response rate of only 11–15%; therefore, this type of tumor typically has a poor prognosis, particularly in late stages. The survival rates of patients with CCC are significantly lower than those of patients with serous EOC [6]. Identifying novel therapeutic targets and establishing new treatment strategies for CCC is thus important.

The common molecular genetic alterations identified so far in CCC include mutations in *ARID1A* and *PI3K* as well as HNF1B overexpression. However, the molecular landscape of CCC oncogenesis remains poorly understood [7, 8]. Since chromosomal aberrations are a cardinal feature of carcinogenesis, the identification of amplified or deleted chromosomal regions associated with CCC would elucidate its underlying pathogenetic mechanisms. Amplification at chromosome17q23-25 has been reported to occur with a frequency of approximately 40% in CCC [9]. The *PPM1D* gene (also known as *WIP1*) maps to the 17q23.2 amplicon and is amplified and/or overexpressed in various types of cancers, including CCC [10]. However, the frequency of *PPM1D* overexpression in CCC is reported to be only about 10%. In addition, the peak region of 17q23-25 amplification in CCC as assessed by GISTIC analysis maps adjacent to the *PPM1D* locus. Taken together, these findings suggest the involvement of undiscovered driver genes on 17q23-25 in CCC [11].

Recent evidence has shown that microRNAs (miRNAs) can have oncogenic or tumor suppressor functions and contribute to cancer biology [12, 13]. Aberrant expression of miRNAs has been shown to be associated with oncogenesis. One of the most frequently overexpressed miRNAs in many types of cancers is *miRNA-21*, located on 17q23.2 within the intron of the *TMEM49* gene [14]. Protein expression of the *PTEN* gene, a target gene of *miR-21*[15], is absent in one-third of all CCC cases [16, 17]. We thus hypothesized that *miR-21* is a potential candidate for 17q23-25 amplification and might play an important role in CCC oncogenesis through the regulation of PTEN expression.

## Methods

### Clinical specimens and ovarian cancer cell cultures

Tissue specimens were obtained from 28 patients with ovarian CCC who were treated at Jikei University Hospital from 2000 to 2010. The Jikei University School of Medicine Ethics Review Committee approved the study protocol (ethics approval number: 14-132) and informed consent was obtained from all patients. Most patients (27 of 28) underwent surgical resection followed by adjuvant chemotherapy with platinum-based regimens (platinum/paclitaxel, n = 12; platinum/irinotecan hydrochloride, n = 13; docetaxel/carboplatin, n = 2) as initial treatment. None of the patients had received chemotherapy or radiation therapy before the initial surgery. All samples were examined as hematoxylin–eosin-stained sections by a pathologist to confirm pure CCC histologically. Tumors were classified according to the World Health Organization classification system, and clinical stages were determined using the International Federation of Gynecology and Obstetrics (FIGO) staging system. Progression-free survival (PFS) was defined as the time from the date of primary surgery to the date of disease progression. Overall survival (OS) was calculated for the time from the date of initial surgery to the last follow-up visit or death. The mean age was 53 years (range, 37–81). FIGO staging was as follows: Stage I, n = 18; stage II, n = 2; stage III, n = 8. The median follow-up period was 45.7 months (range, 5.1–99.3). Coexistent endometriosis was found in 20 (71.4%) of 28 patients. The ovarian CCC cell lines JHOC-5 and JHOC-9 were obtained from Riken Bioresource center (Tsukuba, Japan). HAC-2 was kindly provided by Dr. Nishida (Tsukuba University, Ibaraki, Japan). RMG-I and RMG-II were provided by Dr. D. Aoki (Keio University, Tokyo, Japan). HAC-2, JHOC-5, and JHOC-9 cells were cultured in RPMI-1640 medium (Sigma-Aldrich, Tokyo, Japan). RMG-I and RMG-II were cultured in Ham F-12 medium (Sigma-Aldrich). Both media contained 10% heat inactivated fetal bovine serum, Penicillin-Streptomycin-Amphotericin B Suspension (×100) (Wako, Osaka, Japan). Cells were incubated at 37°C in a humidified atmosphere containing 5% CO_2_.

### DNA and RNA isolation

All surgical samples were composed of at least 80% neoplastic cells and were immediately frozen after collection. For RNA isolation, the fresh clinical specimens were stored at 4°C for 24 hours in RNAlater (Ambion, Austin, Texas, USA) and were then frozen at −80°C in liquid nitrogen until further use. Using a commercially available DNA isolation kit (GentraPureGene kit; Qiagen, Tokyo, Japan), genomic DNA was extracted from stored frozen tumor samples following the manufacturer's instructions. Total RNA was isolated from tumor samples and cell lines with Trizol reagent (Invitrogen, Carlsbad, CA, USA), according to the manufacturer’s instructions. Total RNA from the tumor samples was stored in RNAlater.

### Candidate gene selection

#### Array comparative genomic hybridization (aCGH)

For this validation study, aCGH was performed using the Agilent Human Genome CGH 244AMicroarray Kit 244 K (Agilent Technologies, Santa Clara, CA, USA). DNA digestion, labeling, and hybridization were performed as recommended by the manufacturer. The test DNA (2 μg) and reference DNA (2 μg) were digested with Rsa I and Alu I (Promega). The digested tumor DNA and reference DNA were labeled with either cyanine (Cy) 5-deoxyuridine triphosphate (dUTP) or Cy3-dUTP using the Agilent Genomic DNA Labeling Kit PLUS (Agilent Technologies). Labeled DNAs were purified using Microcon YM-30 filters (Millipore, Billerica, MA, USA). The hybridization mixture, containing Cy3-labeled test DNA and Cy5-labeled reference DNA, 2× Hybridization buffer (Agilent), 10× blocking agent (Agilent), and Human Cot-1 DNA (Invitrogen), was prepared in an Agilent SureHyb chamber. All microarray slides were scanned on the Agilent Microarray Scanner G2505B. Date was obtained using Feature Extraction software, version 10.7.3.1 (Agilent Technologies). Penetrance of aberrant chromosomal areas across the genome was demonstrated using Aberration Detection Method 2 (Agilent Genomic Workbench Lite Edition 6.5.0.18, Agilent Technologies), a quality-weighted interval score algorithm that identifies aberrant intervals in samples that have consistent gain or loss log ratios based on their statistical score. The log_2_ ratios for whole chromosomal number changes that were completely gained, lost, or had no change were evaluated. The threshold for determining amplification or deletion was defined as log_2_ ratio >0.5 or < −0.5.

#### Copy number assay for region 17q23–25 in the miR21 gene in CCC cells

The copy number for the 17q23–25 region was determined using commercially available and custom TaqMan Copy Number Assays (Applied Biosystems, Foster City, CA, USA). The *TERT* locus was used as an internal reference copy number. Genomic DNA was extracted from CCC cell lines (HAC-2, JHOC-5, JHOC-9, RMG-I, and RMG-II) using commercially available gDNA extraction and purification kits. Real-time genomic PCR was performed in a total volume of 20 μL per well containing TaqMan genotyping master mix (10 μL), genomic DNA (20 ng), and primers (20 ng each). Data were analyzed using SDS 2.2 sand CopyCaller software (Applied Biosystems).Copy numbers were assigned as follows: actual copy number <0.5, assigned copy number 0 (gene deletion); actual copy number ≥0.5 but <1.5, assigned copy number 1; actual copy number ≥1.5 but <2.5 , assigned copy number 2; actual copy number ≥2.5 but <3.5, and assigned copy number 3.

### Quantitative reverse transcription-polymerase chain reaction

Reverse transcription (RT) of *miR-21* was carried out using the Taqman microRNA reverse transcription kit (Applied Biosystems, Foster City, CA, USA). cDNAs were synthesized from 2 μg of total RNA using the Superscript cDNA Synthesis Kit (Invitrogen) for *PPM1D* and *PTEN* mRNA detection. Real-time PCR Reactions with TaqMan Fast Advanced Master Mix (Applied Biosystems) were performed in 96-well plates using the Applied Biosystems StepOnePlus Real-time PCR System (Applied Biosystems). Each reaction was analyzed in triplicate. *MiR-21* expression was normalized to that of *U6* small nuclear RNA, and *PPM1D* and *PTEN* expression was normalized to that of *GAPDH*. The expression of *miR-21*, *PPM1D*, and *PTEN* were defined based on the threshold cycle (Ct); relative expression levels are presented as 2^–ΔΔCt^.

### Immunohistochemical analysis

Immunohistochemical analysis of PTEN expression (1:100 dilution, Cell Signaling Technologies) was performed on 3-μm paraffin sections of formalin-fixed, paraffin-embedded tissues using the Ventana Discovery XT automated stainer (Ventana Medical Systems, Tucson, AZ, USA). After deparaffinization, antigen retrieval was carried out in CC1 buffer (Cell Conditioning 1; citrate buffer pH 6.0, Ventana Medical Systems). PTEN expression was scored independently by two investigators (Y. H. and N. Y.) based on stain intensity and extent. Immunohistochemical scoring was conducted in a manner entirely blinded to all clinical and biological variables. The intensity of positive staining was scored from 0 to 2 as follows: 0 (none), 1 (weak; intensity < positive control), 2 (strong; intensity ≥ positive control). Positive staining was assigned using a semi-quantitative, five-category grading system: 0, <5% positive cells; 1, 6–25% positive cells; 2, 26–50% positive cells; 3, 51–75% positive cells; 4, 76–100% positive cells. Addition of the two values gives the total score, and a score <4 was considered PTEN-negative.

### Additional cohort

Additional cohort study was also approved by The Jikei University School of Medicine Ethics Review Committee (ethics approval number: 14-132). An additional cohort was analyzed using aCGH, realtime-PCR, and immunohistochemistry. This additional cohort was included to ensure association between miR21 overexpression and PTEN protein loss using 43 patients, with further confirmation in an additional 15 patients.

### Western blot analysis

Western blot analysis was performed to detect PTEN protein expression (dilution of 1:2000, Cell Signaling Technologies, Danvers, MA, USA). CCC cell lines were washed in PBS and lysed in RIPA buffer containing 200 mM Tris-HCl (pH 7.2), 150 mM NaCl, 0.1% SDS, 1% Nonidet P-40, 1% sodium deoxycholate, 2 mM EDTA, 50 mM NaF, 1% proteinase inhibitors, and 1% PMSF for 10 min on ice. Cell lysates were then sonicated for 30 seconds, and cellular debris were removed by centrifugation at 14 000 rpm at 4°C for 30 min. Supernatants were collected and assayed for protein concentration using the BCA Protein Assay Kit (Invitrogen). Supernatants containing an equal amount of protein extract were supplemented with concentrated 4× LDS sample buffer (Invitrogen) and heated at 95°C for 5 min. Approximately 40 μg of lysate was loaded onto a 12.5% SDS-polyacrylamide gel. The supernatants were separated by SDS–PAGE, and proteins were transferred to Immobilon-P transfer membrane (Millipore, Milford, MA, USA). The transfer membrane was incubated with primary antibody in TBS with 0.1% Tween-20 and 5% bovine serum albumin overnight at 4°C. Anti-rabbit IgG-conjugated horseradish peroxidase (GE Healthcare) was used as the secondary antibody. The transfer membrane was incubated with secondary antibody in TBS with 0.1% Tween-20 and 5% skim milk for 90 min at room temperature. The proteins were visualized using the ECL-Plus Western blotting detection system and detected using the Image Quant LAS 4000 mini (GE Healthcare). The concentration of each target protein was normalized against beta-actin.

### Transfection

Twenty four hours before transfection, cells were seeded in plates and grown to 50% confluence. For inhibition of miR-21, RMG-II cells were transfected with *mir*Vana miRNA Inhibitors or a control (Ambion). Transfections were performed using Lipofectamine RNAiMAX (Invitrogen) according to the manufacturer’s protocol.

### Dual luciferase reporter assay

pGL3 wild-type *PTEN* 3′-UTR and pGL3 mutant-type *PTEN* 3′-UTR luciferase plasmids were obtained from Addgene (Cambridge, MA). RMG-II cells were seeded in 6-well plates (5×105 cells/well). After 24 h, the cells were transfected with pGL3 control vector (Promega), pGL3 wild-type *PTEN* 3′-UTR vectors, or pGL3 mutant-type *PTEN* 3′-UTR vectors using Lipofectamine 2000 reagent. Luciferase activities were measured using the Dual-Luciferase Reporter Assay system (Promega) 24 h after transfection. Firefly luciferase activity was normalized to renilla activity for each sample. All the experiments were performed in triplicate.

### MTS assay

MTS assay was performed using the CellTiter 96 AQueous One Solution Cell Proliferation Assay kit (Promega, Madison, WI, USA) following the manufacturer's protocol. Briefly, miR-21 inhibitor and negative control oligonucleotides were transfected at a final concentration of 200nM. After 24 hours transfection, RMG-II cells were seeded into 96-well plates at a density of 1 × 10^4^ cells per well. MTS (20 μL) was added to each well 3 hours before the desired time points, and cells were incubated at 37°C. The absorbance was measured at 490 nm using a Microplate Reader (VersaMAx, Molecular Devices). All experiments were repeated three times. Values are presented as the mean ± standard deviation (SD).

### Invasion assay

Cells were seeded into the top chamber of a 96-well matrigel-coated plate with 8-μm-pore polyethylene terephthalate membrane inserts (Corning). MiR-21 inhibitor and negative control oligonucleotides were transfected at a final concentration of 200nM.The bottom chamber was filled with 0.75 mL Ham F-12 medium with 10% FBS as a chemoattractant. The inserts were filled with 0.5 mL Ham F-12 medium with 1% FBS. After incubation for 48 h, the filter membrane was fixed with 100% methanol and stained with hematoxylin and eosin. The degree of invasiveness was quantified by counting the number of cells in 4 random fields of view per filter using 400× magnification. Data obtained from three separate inserts are shown as mean values.

### Statistical analysis

All statistical analyses were performed using StatMate III software (ATMS, Tokyo, Japan). Comparisons between parameters were made using Fisher’s exact test. For survival analysis, PFS and OS distributions were determined using the Kaplan–Meier method, and the resulting curves were compared using the log-rank test. P <0.05 was considered statistically significant.

## Results

### Chromosome 17q23-25 amplification, miR-21 expression, and PTEN protein expression in CCC

**Figure 1:**
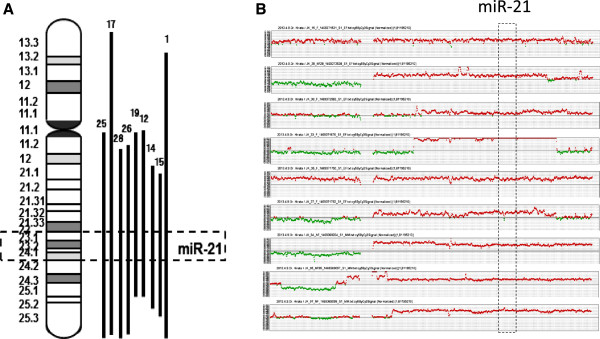
**Frequency of copy number changes in chromosome 17 by array CGH in 28 CCC. (A)** chromosome 17 is represented by ideograms showing G-banding patterns. Bold vertical lines on the ideogram indicate the region of chromosomal amplification. The number at the top of each line represents the primary tumor in which the indicated change was recorded. Nine samples showed 17q23-25 amplification that included miR-21. **(B)** The gains and losses are shown as green and red color bars, respectively. These samples showed 17q23-25 amplification that included miR-21.

**Figure 2:**
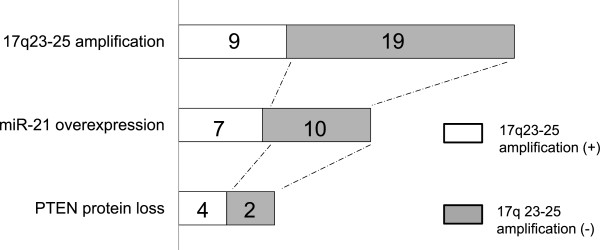
**Analysis of clinical CCC specimens.** Of the 9 tumors with 17q23-25 amplification, 7 (77.7%) showed *miR-21* overexpression. Of the 19 tumors (58.8%) without 17q23-25 amplification, 10 showed *miR-21* overexpression. Of all the 28 17q23-25 amplification cases, both *miR-21* overexpression and PTEN protein loss were detected in 4 (14.2%).

CGH array profiles of chromosome 17 in 28 primary CCCs revealed that 9 out of 28 patients (32%) showed 17q23-25 amplification that included *miR-21* (Figure 1). *MiR-21* and *PPM1D* mRNA expression were then measured by real-time RT-PCR analysis (Additional file 1: Figure S1). We defined standardized value as each median value of *miR-21* and *PPM1D* expression without 17q23-25 amplification. Overexpression of *miR-21* and *PPM1D* were found in 60% and 57% of these tumors, respectively. Seven of 9 tumors (77.7%) with 17q23-25 amplification showed *miR-21* overexpression, and 10 of 19 tumors (52.6%) without 17q23-25 amplification also showed *miR-21* overexpression. In addition, 6 of 9 tumors (66.6%) with 17q23-25 amplification showed *PPM1D* overexpression, and 10 of 19 tumors (52%) without 17q23-25 amplification showed *PPM1D* overexpression (Additional file 1: Figure S1). We next evaluated the relationship between 17q23-25 amplification and either *miR-21* or *PPM1D* overexpression. No significant correlation between the amplification and overexpression was observed for either gene. Next, immunohistochemical analysis of PTEN (a potential target of *miR-21*) was performed on samples from the same primary CCC patients. Loss of PTEN protein was observed in 13 of 28 patients (46.4%) (Additional file 2: Figure S2) and in 6 of 17 tumors (35.3%) with *miR-21* overexpression. No significant correlation was observed between *miR-21* overexpression and loss of PTEN expression. To further confirm these results, we added 15 CCC samples from an additional cohort, performing real-time RT-PCR of miR21 and IHC of PTEN. Again, no significant correlation was observed between *miR-21* overexpression and loss of PTEN expression (date not shown). In total, as shown in Figure 2, the occurrence of 17q23-25 amplification with both *miR-21* overexpression and PTEN protein loss was detected in 4 out of 28 CCC patients (14.2%) (Figure 2).

### Associations between clinicopathological parameters and either 17q23-25 amplification, miR-21 overexpression, or PTEN protein loss

The relationship between clinicopathological parameters and genetic alterations including 17q23-25 amplification, *miR-21* overexpression, and decreased PTEN protein expression are summarized in Table 1. Interestingly, a significant correlation was observed between *miR-21* overexpression and endometriosis. Meanwhile, no correlations were observed between the other clinical parameters and any of the genetic alterations. According to survival analysis, patients with 17q23-25 amplification had significantly shorter progression-free and overall survival times than did those without 17q23-25 amplification (log-rank test; PFS, p = 0.0496; OS, p = 0.0469) (Table 2). On the other hand, the PFS and OS did not correlate significantly with *miR-21* overexpression or PTEN protein loss.

**Table 1:** **Associations between clinicopathological parameters and either 17q23-25 amplification,** ***miR-21*** **overexpression, or PTEN protein loss**

Variable	Cases (Total 28)	17q23-25 amplification		***miR-21*** overexpression		Loss of PTEN protein expression	
		n = 9	P value	n = 17	P value	n = 13	P value
Age	6			4	>0.9999	5	0.0690
≧60	22	2	>0.9999	13		8	
< 60		7					
Stage			0.6464		>0.9999	8	0.1977
I-II	21	6		11		5	
III-IV	7	3		4			
Lymph node status	7				>0.9999	4	0.6702
Metastasis	21	3	0.6219	4		9	
No metastasis		6		11			
Endometriosis		8	0.2143	15		10	0.6859
Positive	20	1		2	0.0298	3	
Negative	8						
Thrombosis	3	1	>0.9999	2	>0.9999	2	0.5833
Positive	25	8		13		11	
Negative							

No correlations were observed between the other clinical parameters (age, stage, lymph node metastasis, thrombosis, and either 17q23-25 amplification, *miR-21* overexpression, or PTEN protein loss). A significant correlation was observed between *miR-21* overexpression and endometriosis. P-values were from two-sided tests and statistically significant when <0.05.

**Table 2:** **Proportional hazard regression analysis of single predictors for PFS and OS in CCC**

Parameters	PFS		OS	
	95%CI	P-value	95%CI	P-value
Age (≦60 vs. >60 years)	0.289–1.656	0.3371	0.244-–1.965	0.3337
Stage (I, II vs. III, IV)	0.289–1.234	<0.05	0.289–1.168	<0.05
Endometriosis	0.153–2.834	0.2384	0.154–2.684	0.2156
Residual tumor ≦2 VS. >2 cm)	0.3440–2.484	<0.05	0.1332–2.408	<0.05
17q23-25 amplification	0.1768–1.684	0.0496	0.154–1.756	0.0469
*miR-21* overexpression	0.441–1.168	0.3141	0.441–1.645	0.3204
PTEN protein loss	0.4422–1.980	0.6393	0.3771–1.465	0.7067

PFS, progression-free survival; OS, Overall survival; CI, Confidence interval.

For survival analysis, PFS and OS distribution was determined using the Kaplan–Meier method. The patients with 17q23-25 amplification had significantly shorter PFS and OS than that did those without 17q23-25 amplification in CCC tumors Meanwhile, PFS and OS did not show significant correlations in *miR-21* overexpression, PTEN protein loss, or clinicopathological date.

### MiR-21 modulates PTEN expression

Based on the profiles of 17q23-25 copy number changes, *miR-21* expression*, PTEN* mRNA expression, and PTEN protein expression in 5 CCC cell lines, we selected RMG-II cells for further functional analysis. We considered this cell line to be ideal because the cells showed relatively 17q23-25 amplification, high *miR-21* expression with decreased PTEN protein expression (Additional file 3: Figure S3 and Additional file 4: Figure S4).

**Figure 3:**
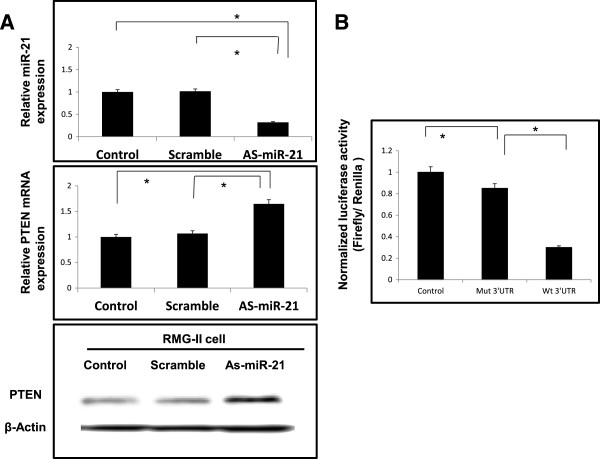
***miR-21*** **modulates PTEN tumor suppressor gene expression.** To evaluate the biological significance of *miR-21* overexpression in CCC, we used a loss-of-function antisense approach. An antisense *miR-21* oligonucleotide (ODN) was used to knock down *miR-21* expression in RMG-II cells. **(A)** Efficiency of RMG-II cell transfection was confirmed by real- time RT PCR. *PTEN* mRNA expression was increased by knockdown of *miR-21* in RMG-II cells. Western blot analysis showing that PTEN expression was increased in RMG-II cells upon inhibition of *miR-21*. **(B)** *MiR-21* directly targets the 3'-UTR of *PTEN* mRNA*.* The activity of luciferase in the pGL3 wild-type *PTEN* 3′-UTR was downregulated compared to pGL3 mutant-type *PTEN* 3’-UTR and the pGL3 control in RMG-II cells. P <0.05 according to the t-test.

To investigate the regulation of PTEN expression by *miR-21* in CCC, we used a loss-of-function antisense approach in RMG-II cells. Knockdown efficiency was confirmed by real-time RT-PCR analysis of *miR-21* (Figure 3A). In RMG-II cells, we found that *miR-21* knockdown caused a significant increase in PTEN protein expression as indicated by Western blot analysis, along with increased *PTEN* mRNA expression (Figure 3A). However, suppression of *miR-21* expression did not inhibit cell proliferation or invasion (date not shown). We next investigated the direct binding of *miR-21* to the 3’UTR of PTEN mRNA by luciferase assay using a pGL3 plasmid harboring either the wild- or mutant-type *PTEN* 3’-UTR. The activity of the luciferase reporter was significantly decreased when fused to the wild-type *PTEN* 3′-UTR. Deletion mutations in the *miR-21*–interacting seed region rescued the luciferase activity. Taken together, these data suggest that *PTEN* is a direct functional target of *miR-21*, and its expression is regulated by *miR-21* in CCC (Figure 3B). Several potential miR21 targets that could have implications in CCC were identified using web-based computational approaches to predict gene targets (miRBase Targets BETA Version 1.0, PicTar predictions, and TargetScan). Three putative target genes, PDCD4, SMARCA4, and SPRY2, were predicted by 3 different programs. This result indicates that tumor suppressor genes are potentially regulated by miR21. Therefore, we performed real-time RT-PCR for PDCD4, SMARCA4, SPRY2 in the miR21 knockdown experiments in RMG-II cells. We found that *miR-21* knockdown increased the expression of these mRNAs (Additional file 5: Figure S5). To investigate the regulation of PTEN expression by *miR-21* in JHOC9 cells, we overexpressed miR21 using miR21 mimics in JHOC9 cell. Quantitative real-time PCR analysis confirmed the level of miR21 was significantly overexpressed. As expected, the level of PTEN mRNA was downregulated in JHOC9 cells. Expression of PDCD4, SMARCA4, and SPRY2 mRNA was also decreased by the overexpression of miR-21 in response to miR-21 mimics in JHOC9 cells (Additional file 6: Figure S6).

## Discussion

DNA copy number aberrations are a frequent event in many malignant tumors, leading to altered expression and function of genes residing within the affected genome region. Such genomic abnormalities can harbor either oncogenes or tumor suppressor genes depending on the original gene function and whether the copy number is amplified or deleted. Previous studies have identified a high frequency of copy number amplifications in CCC, including 17q23-25 (18-40%), 20q13 (22-25%), and 8q21q- 24q. Additionally, deletions at chromosome 9q and 19p have been also reported in CCC [9, 18–20]. Of the chromosomal alterations associated with CCC, 17q23-25 is one of the most frequently amplified regions and is reported to be associated with patient outcome [9]. So far, *PPM1D* and *APPBP2* have been identified as potential targets of 17q23-25 amplification in CCC. However, a recent report suggests there might be new driver genes other than *PPM1D* and *APPBP2* in this region [11]. More than half of miRNAs have been aligned to genomic fragile sites or frequently deleted or amplified regions in several malignancies [21, 22]. MiRNAs are a class of small, non-coding RNA molecules that regulate gene expression through translational repression or cleavage of target mRNA. Among them, *miR-21*, located on 17q23.2, is unique in that it is overexpressed in many cancers as an oncogene. Previous studies have revealed several significant *miR-21* targets that might be related to carcinogenesis. Based on this evidence, *miR-21* is a potential candidate for 17q23-25 amplification in CCC oncogenesis.

We analyzed DNA copy number alterations at chromosome 17 in a panel of 28 primary CCCs using CGH array. In our data set, 17q23-25 amplification was observed at a frequency similar to that of previous reports. In addition, we confirmed that 17q23-25 amplification correlated negatively with patient prognosis, suggesting that the chromosomal alteration might result in the overexpression of genes that contribute to the genomic instability of CCC. Although we did not find a statistical correlation between *miR-21* overexpression and amplification of this region, overexpression of *miR-21* was observed in 60% of the CCC cases examined.

Targets of *miR-21* in cancer include *PTEN*, *PDCD4*, *LRRFIP1*, *RECK*, *TIMP-3*, *TPM1*, *BTG2*, and *Sprty2*[23]. *PTEN* can restrict growth and survival signals by limiting the activity of the phosphoinositide 3-kinase (PI3K) pathway. A decrease in PTEN might cause activation of the PI3K pathway, including Akt and mTOR, which leads to tumor development [24]. The prominent role of PTEN inactivation in CCC is thought to involve multiple mechanisms. In our study, loss of PTEN protein was observed in 46% of CCC patients. On the other hand, low of PTEN copy number was not indicted by CGH array (data not shown). Furthermore, no significant correlation was observed between *miR-21* overexpression and loss of PTEN expression in our date set. Therefore, we suggest the involvement of another epigenetic mechanism, such as *PTEN* mutations, promoter methylation of *PTEN*, loss of heterozygosity at the *PTEN* locus other miR are infrequent in CCC. Although there was no statistical correlation between PTEN loss and *miR-21* overexpression, the occurrence of 17q23-25 amplification along with both *miR-21* overexpression and PTEN protein loss was detected in 14% of CCC cases. Thus, this oncogenetic mechanism might play a prominent role in CCC. Additionally, we showed that *miR-21* inhibition significantly increased PTEN expression in vitro. Moreover, the results obtained from the dual luciferase reporter assay supports the idea that *miR-21* directly targets the *PTEN* gene, regulating the protein expression. It is therefore possible that miRNAs such as *miR-21* modulate PTEN expression by transcriptional regulation or target degradation in CCC.

Finally, we found a significant correlation between *miR-21* overexpression and endometriosis in CCC. Endometriosis-related CCC is thought to be a chronic inflammatory disease, characterized by increased production of pro-inflammatory cytokines such as IL-1, IL-6, IL-8, IL-10, and TNF-α [25]. We recently reported that CCC showed a dominant Th-2 cytokine expression pattern driven largely by *IL-6* expression [26]. In addition, IL-6 induces *miR-21* expression through a STAT3-dependent pathway [27]. We also confirmed that IL-6 induces *miR-21* overexpression in RMG-II (data not shown). In our study, *miR-21* overexpression was observed in 60% of the CCC cases, regardless of 17q23-25 amplification status, suggesting another mechanism might regulate *miR-21* expression. *miR-21* might contribute to inflammation-induced carcinogenesis in CCC with endometriosis. We need to further analyze miR21 expression using in situ hybridization in the endometriotic lesions of CCC specimens. The correlation between miR21 and endometriosis observed in our study indicates a role for miR21 in precursor lesions of ovarian CCC.

## Conclusions

**Figure 4:**
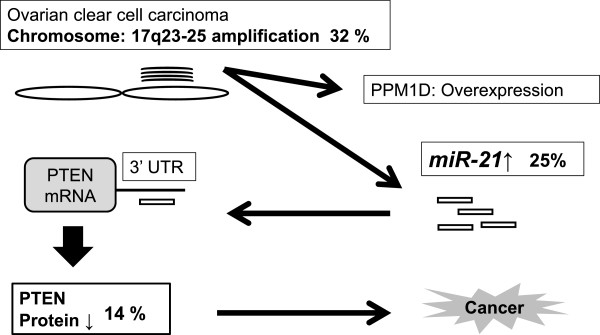
**Chromosome 17q23-25 amplification,** ***miR-21*** **expression, and PTEN protein expression in CCC.** CGH array was performed to evaluate chromosomal alterations in 28 primary CCC tumors. Nine out of 28 patients (32%) showed chromosomal amplification in the 17q23-25 region that contains *miR-21*. Seven of 9 tumors (77.7%) with 17q23-25 amplification showed *miR-21* overexpression. 17q23-25 amplification with both *miR-21* overexpression and PTEN protein loss was detected in 4/28 cases (14.2%).

This study is the first to indicate *miR-21* as the gene of interest in 17q23-25 amplification associated with CCC (Figure 4). Aberrant expression of *miR-21* by chromosomal amplification might play an important role in CCC carcinogenesis through regulating the *PTEN* tumor suppressor gene. Moreover, the modulation by *miR-21* overexpression of genes other than *PTEN* should not be overlooked in determining the oncogenic mechanism of CCC.

## Electronic supplementary material

Additional file 1: Figure S1: *MiR-21* and *PPM1D* mRNA expression located on 17q23- 25. Black dots indicate a cluster with 17q23-25 amplification, and white dots indicate a cluster without 17q23-25 amplification. We measured the median expression of *miR- 21* and *PPM1D* mRNA and set a transverse line as standard value. Seven of 9 tumors with 17q23-25 amplification showed *miR-21* overexpression. Six of 9 tumors with 17q23-25 amplification showed *PPM1D* overexpression. (PPTX 78 KB)

Additional file 2: Figure S2: Immunohistochemical analysis of PTEN that might be a potential target of *miR-21* was performed using the same primary CCC cases. The intensity of positive staining was scored from 0 to 2, while the extent of positive staining was scored from 0 to 4. Addition of the two values gives the total score; scores >4 were considered PTEN-positive. (A) Typical image of a PTEN-negative case. (B) Typical image of a PTEN-positive case. Loss of PTEN protein was observed in 13 of 28 patients (46.4%). (PPTX 2 MB)

Additional file 3: Figure S3: Frequency of copy number changes in Chr 17q23-25 region by copy number assay in 5 CCC cell lines. We found the copy number was increased in RMG-I and RMG-II cells. (PPTX 49 KB)

Additional file 4: Figure S4: *MiR-21*, *PTEN* mRNA, and PTEN protein expression in CCC cell lines. (A) (B) Relative expression of *miR-21* and *PTEN* mRNA were detected with real-time RT-PCR, and the relative amount of *miR-21* was determined using 2-^ΔΔ^CT. (C) PTEN protein was measured by western blotting. The RMG-II cell line was selected for further analysis, because it had the most prominently overexpressed *miR-21* and decreased PTEN protein of the CCC cell lines. (PPTX 58 KB)

Additional file 5: Figure S5: Three putative target genes, PDCD4,SMARCA4, and SRY2, are potentially regulated by miR21. (A) (B) (C) Real-time RT-PCR for PDCD4, SMARCA4, SPRY2 in the miR21 knockdown experiments in RMG-II cells. *miR-21* knockdown caused an increase in mRNA expression of these genes by real-time RT PCR in RMG-II cells. (PPTX 69 KB)

Additional file 6: Figure S6: Mir21 modulates PTEN expression in JHOC9 cell. To investigate the regulation of PTEN expression by *miR-21* in JHOC9 cells, we overexpressed miR21 by miR21 mimics in JHOC9 cells. Quantitative real-time PCR analysis confirmed miR21 was significantly overexpressed. As expected, the level of PTEN mRNA was downregulated in JHOC9 cells. PDCD4, SMARCA4, and SPRY2 mRNAs were also reduced by the overexpression of miR-21 in response to miR-21 mimics in JHOC9 cells. (PPTX 82 KB)
