# Supplementary material for: MicroRNA-21 is a candidate driver gene for 17q23-25 amplification in ovarian clear cell carcinoma
Source: BMC Cancer. 2014 Nov 3;14:799. doi: 10.1186/1471-2407-14-799 (PMC4289307; doi:10.1186/1471-2407-14-799)
Supplement: Supplementary file 1 — Additional file 1: Figure S1: MiR-21 and PPM1D mRNA expression located on 17q23- 25. Black dots indicate a cluster with 17q23-25 amplification, and white dots indicate a cluster without 17q23-25 amplification. We measured the median expression of miR- 21 and PPM1D mRNA and set a transverse line as standard value. Seven of 9 tumors with 17q23-25 amplification showed miR-21 overexpression. Six of 9 tumors with 17q23-25 amplification showed PPM1D overexpression. (PPTX 78 KB) [file 12885_2014_5135_MOESM1_ESM.pptx]

## Slide 1
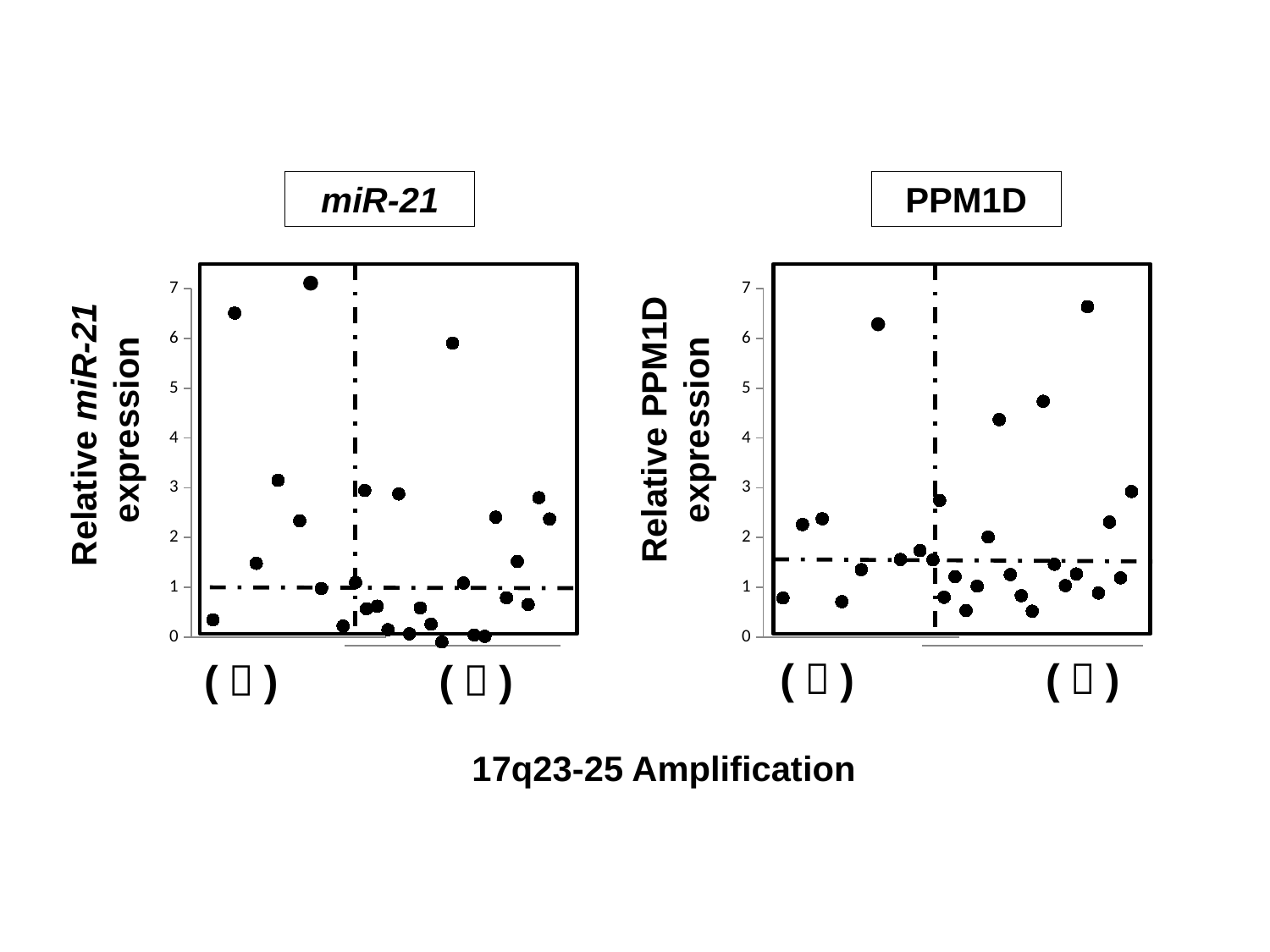

miR-21
PPM1D
### Chart
| Category | |
|---|---|
### Chart
| Category | |
|---|---|
### Chart
| Category | |
|---|---|
### Chart
| Category | |
|---|---|
Relative miR-21
expression
Relative PPM1D
expression
(＋)　 　 　 (－)
(＋) 　 　 (－)
17q23-25 Amplification
