# Supplementary figures and images for: MicroRNA-21 is a candidate driver gene for 17q23-25 amplification in ovarian clear cell carcinoma
Source: BMC Cancer. 2014 Nov 3;14:799. doi: 10.1186/1471-2407-14-799 (PMC4289307; doi:10.1186/1471-2407-14-799)

## Slide 1
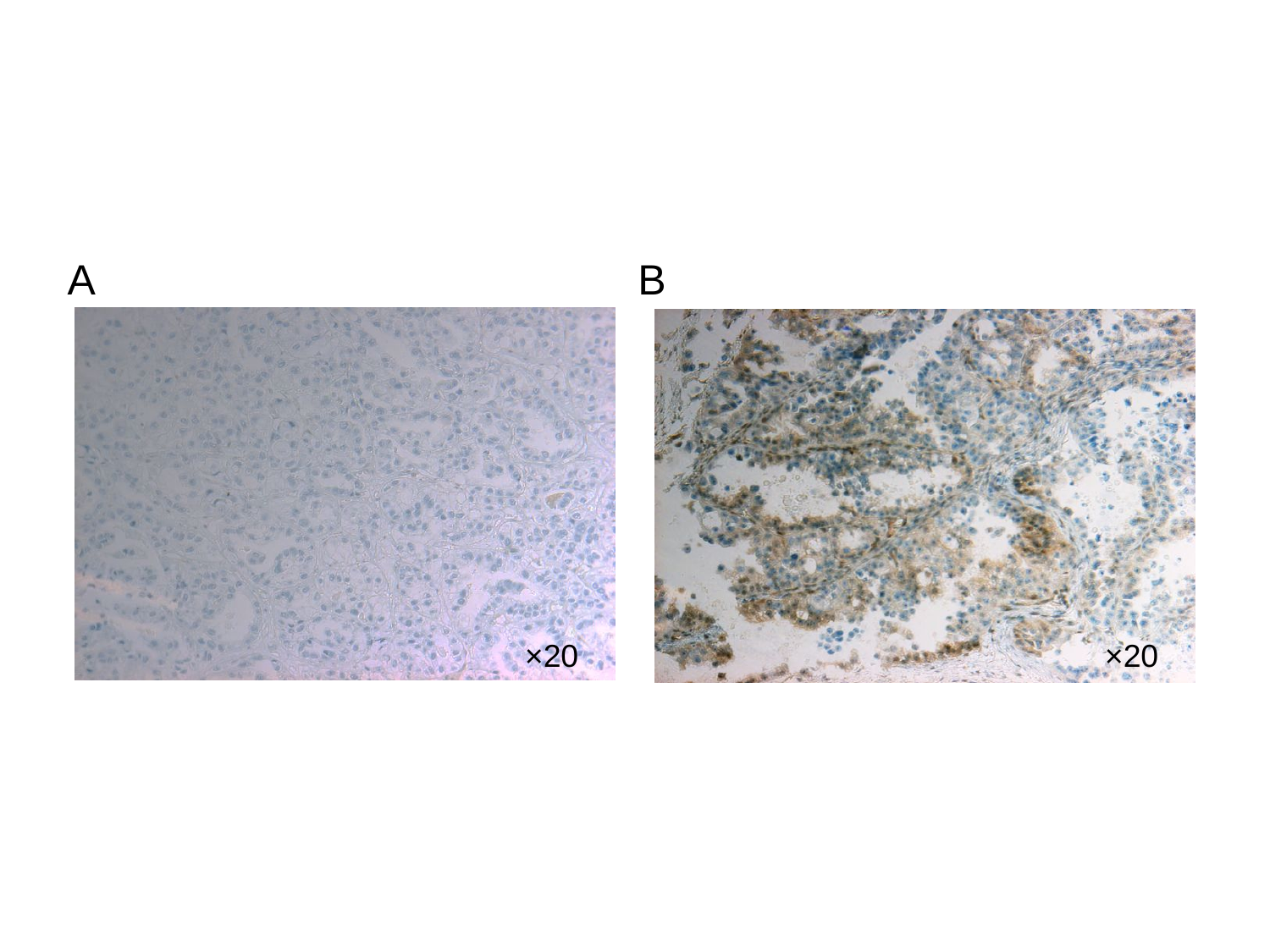

A
B
×20
×20

Supplement: Supplementary file 2 — Additional file 2: Figure S2: Immunohistochemical analysis of PTEN that might be a potential target of miR-21 was performed using the same primary CCC cases. The intensity of positive staining was scored from 0 to 2, while the extent of positive staining was scored from 0 to 4. Addition of the two values gives the total score; scores >4 were considered PTEN-positive. (A) Typical image of a PTEN-negative case. (B) Typical image of a PTEN-positive case. Loss of PTEN protein was observed in 13 of 28 patients (46.4%). (PPTX 2 MB) [file 12885_2014_5135_MOESM2_ESM.pptx]

## Slide 1
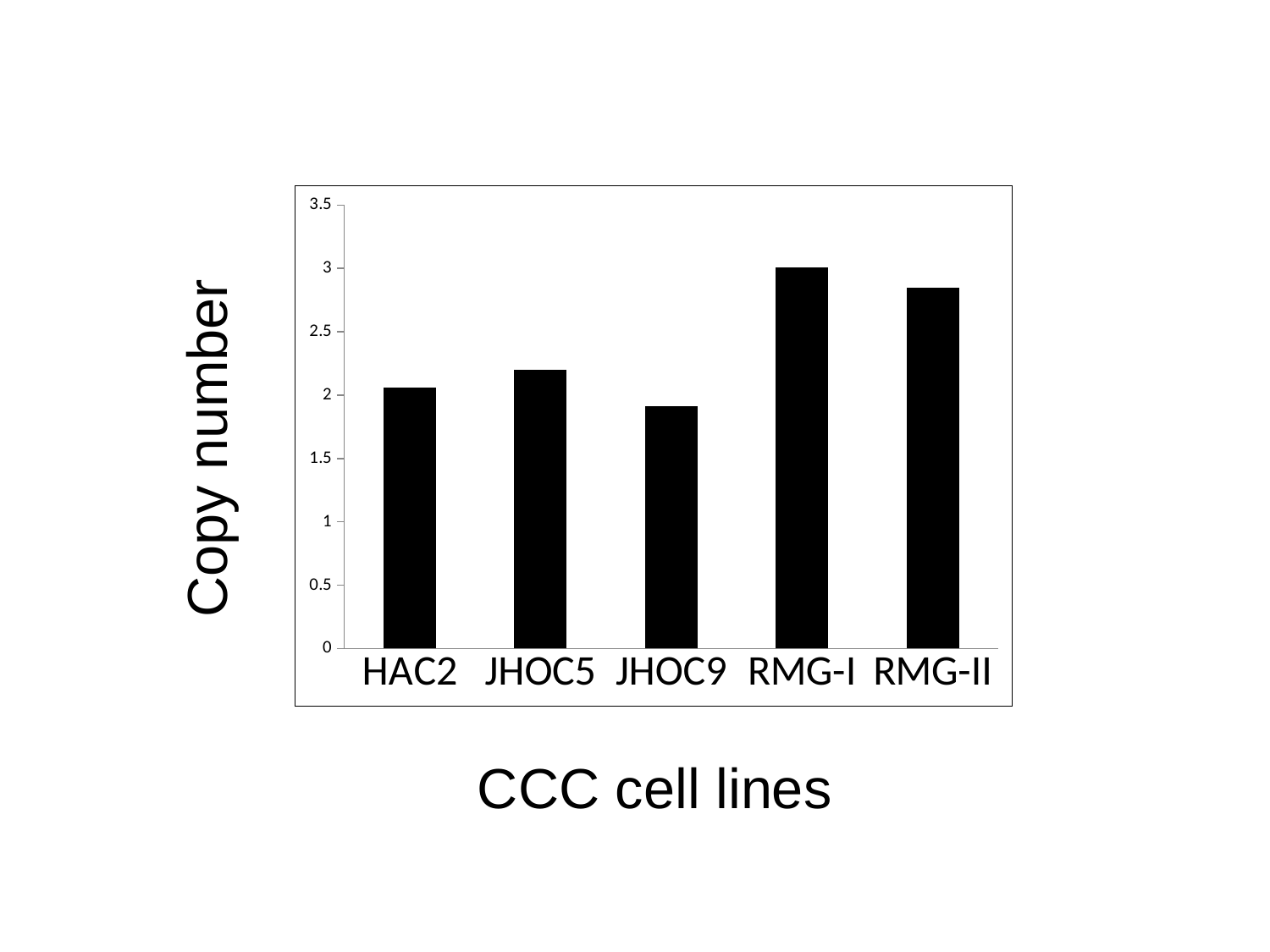

Copy number
### Chart
| Category | |
|---|---|
| HAC2 | 2.06 |
| JHOC5 | 2.2 |
| JHOC9 | 1.91 |
| RMG-I | 3.01 |
| RMG-II | 2.85 |CCC cell lines

Supplement: Supplementary file 3 — Additional file 3: Figure S3: Frequency of copy number changes in Chr 17q23-25 region by copy number assay in 5 CCC cell lines. We found the copy number was increased in RMG-I and RMG-II cells. (PPTX 49 KB) [file 12885_2014_5135_MOESM3_ESM.pptx]
