# Supplementary material for: MicroRNA-21 is a candidate driver gene for 17q23-25 amplification in ovarian clear cell carcinoma
Source: BMC Cancer. 2014 Nov 3;14:799. doi: 10.1186/1471-2407-14-799 (PMC4289307; doi:10.1186/1471-2407-14-799)
Supplement: Supplementary file 4 — Additional file 4: Figure S4: MiR-21, PTEN mRNA, and PTEN protein expression in CCC cell lines. (A) (B) Relative expression of miR-21 and PTEN mRNA were detected with real-time RT-PCR, and the relative amount of miR-21 was determined using 2-ΔΔCT. (C) PTEN protein was measured by western blotting. The RMG-II cell line was selected for further analysis, because it had the most prominently overexpressed miR-21 and decreased PTEN protein of the CCC cell lines. (PPTX 58 KB) [file 12885_2014_5135_MOESM4_ESM.pptx]

## Slide 1
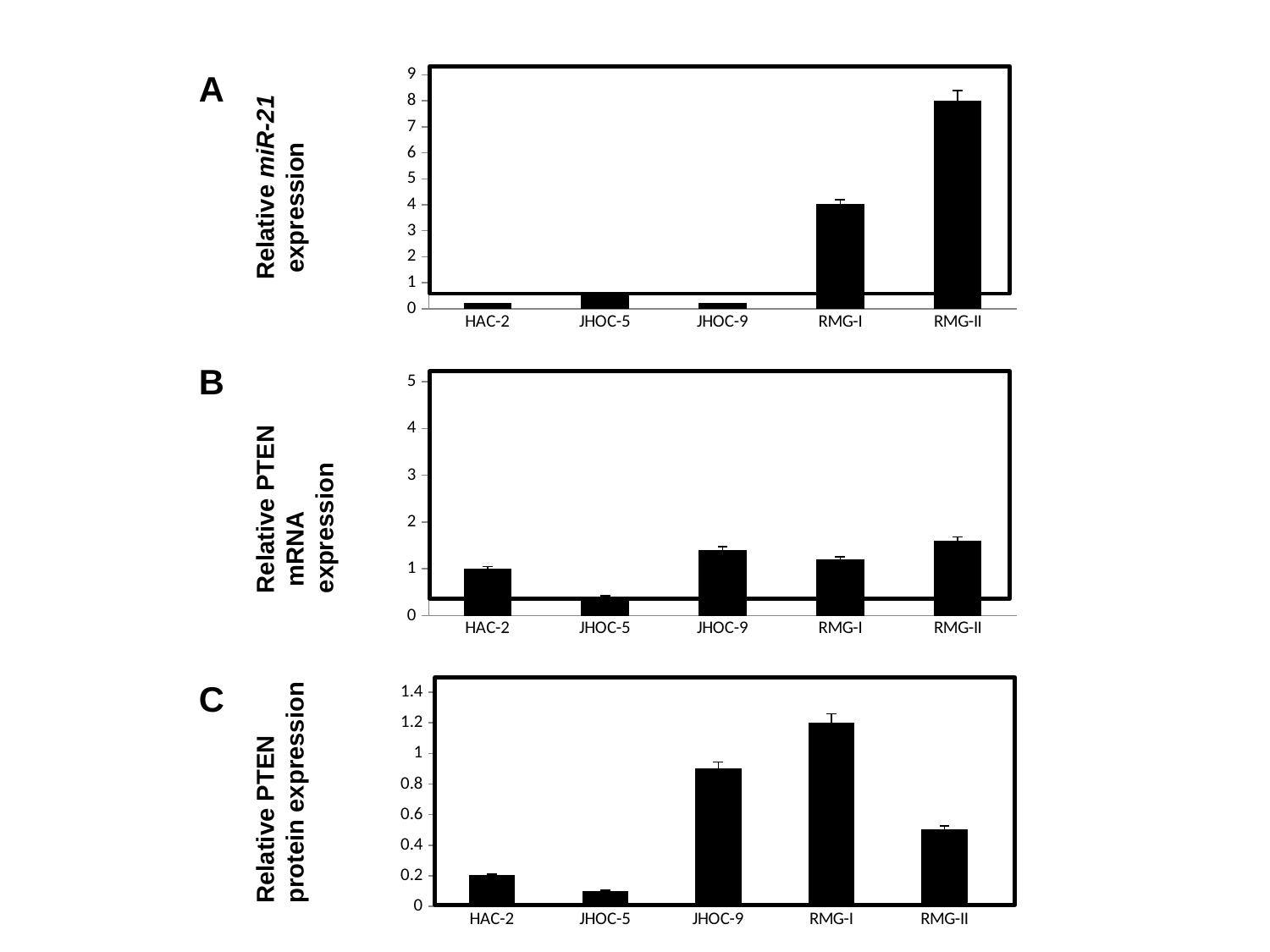

A
Relative miR-21
 expression
Relative PTEN
 mRNA expression
Relative PTEN
protein expression
B
C
### Chart
| Category | |
|---|---|
| HAC-2 | 0.2000000000000001 |
| JHOC-5 | 0.5 |
| JHOC-9 | 0.19999999999999996 |
| RMG-I | 4.0 |
| RMG-II | 8.0 |
### Chart
| Category | |
|---|---|
| HAC-2 | 1.0000000000000004 |
| JHOC-5 | 0.4 |
| JHOC-9 | 1.4 |
| RMG-I | 1.2 |
| RMG-II | 1.6 |
### Chart
| Category | |
|---|---|
| HAC-2 | 0.2000000000000001 |
| JHOC-5 | 0.10000000000000005 |
| JHOC-9 | 0.9000000000000002 |
| RMG-I | 1.2 |
| RMG-II | 0.5 |
