# Supplementary material for: MicroRNA-21 is a candidate driver gene for 17q23-25 amplification in ovarian clear cell carcinoma
Source: BMC Cancer. 2014 Nov 3;14:799. doi: 10.1186/1471-2407-14-799 (PMC4289307; doi:10.1186/1471-2407-14-799)
Supplement: Supplementary file 5 — Additional file 5: Figure S5: Three putative target genes, PDCD4,SMARCA4, and SRY2, are potentially regulated by miR21. (A) (B) (C) Real-time RT-PCR for PDCD4, SMARCA4, SPRY2 in the miR21 knockdown experiments in RMG-II cells. miR-21 knockdown caused an increase in mRNA expression of these genes by real-time RT PCR in RMG-II cells. (PPTX 69 KB) [file 12885_2014_5135_MOESM5_ESM.pptx]

## Slide 1
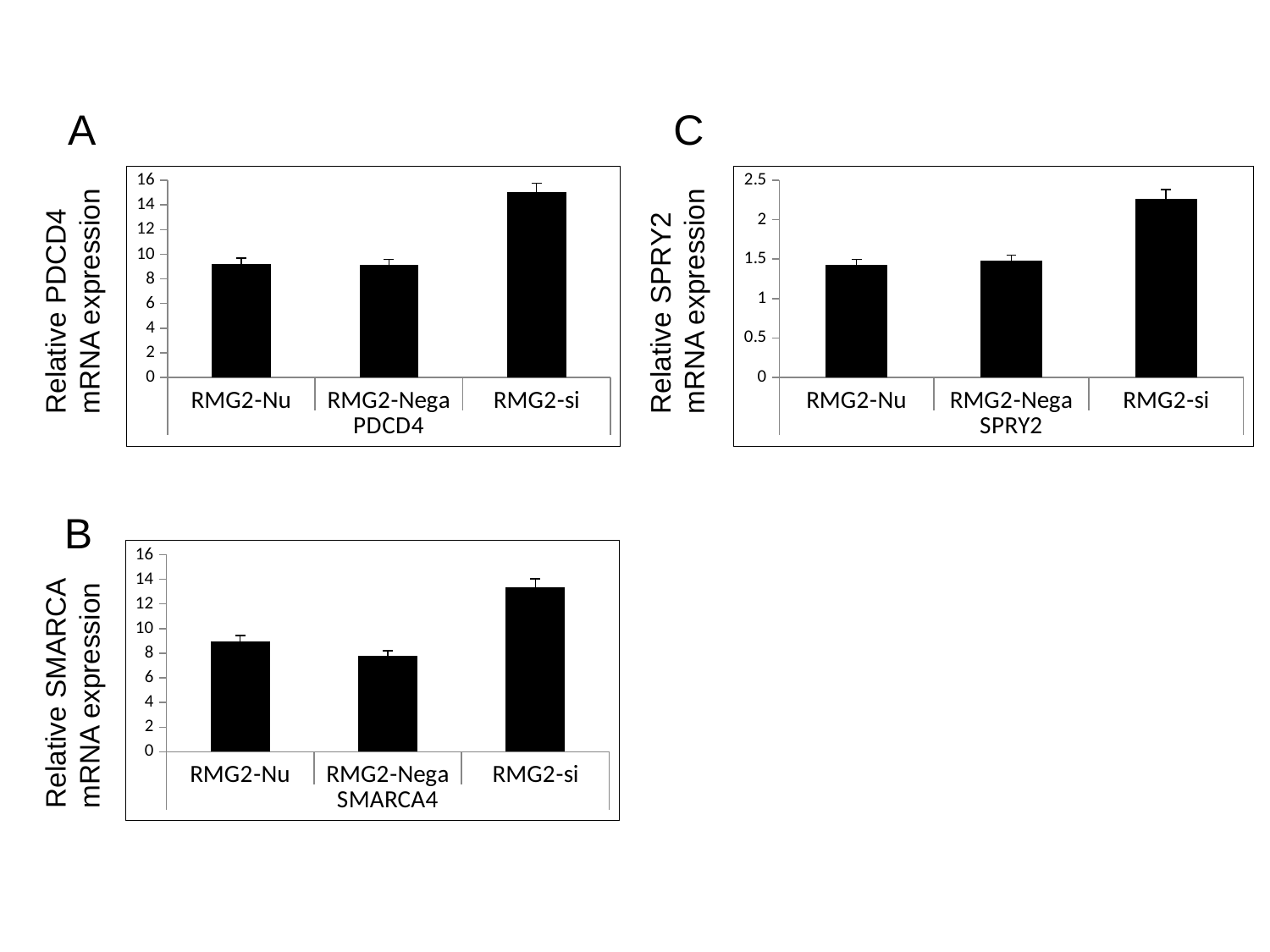

A
C
### Chart
| Category | |
|---|---|
| RMG2-Nu | 9.2346246 |
| RMG2-Nega | 9.14047147790688 |
| RMG2-si | 15.014885752189363 |
### Chart
| Category | |
|---|---|
| RMG2-Nu | 1.4265964 |
| RMG2-Nega | 1.4790519044263628 |
| RMG2-si | 2.26962858871451 |Relative PDCD4
mRNA expression
Relative SPRY2
mRNA expression
B
### Chart
| Category | |
|---|---|
| RMG2-Nu | 8.97980059437095 |
| RMG2-Nega | 7.810176388594978 |
| RMG2-si | 13.383461522128155 |Relative SMARCA
mRNA expression
