# Supplementary material for: MicroRNA-21 is a candidate driver gene for 17q23-25 amplification in ovarian clear cell carcinoma
Source: BMC Cancer. 2014 Nov 3;14:799. doi: 10.1186/1471-2407-14-799 (PMC4289307; doi:10.1186/1471-2407-14-799)
Supplement: Supplementary file 6 — Additional file 6: Figure S6: Mir21 modulates PTEN expression in JHOC9 cell. To investigate the regulation of PTEN expression by miR-21 in JHOC9 cells, we overexpressed miR21 by miR21 mimics in JHOC9 cells. Quantitative real-time PCR analysis confirmed miR21 was significantly overexpressed. As expected, the level of PTEN mRNA was downregulated in JHOC9 cells. PDCD4, SMARCA4, and SPRY2 mRNAs were also reduced by the overexpression of miR-21 in response to miR-21 mimics in JHOC9 cells. (PPTX 82 KB) [file 12885_2014_5135_MOESM6_ESM.pptx]

## Slide 1
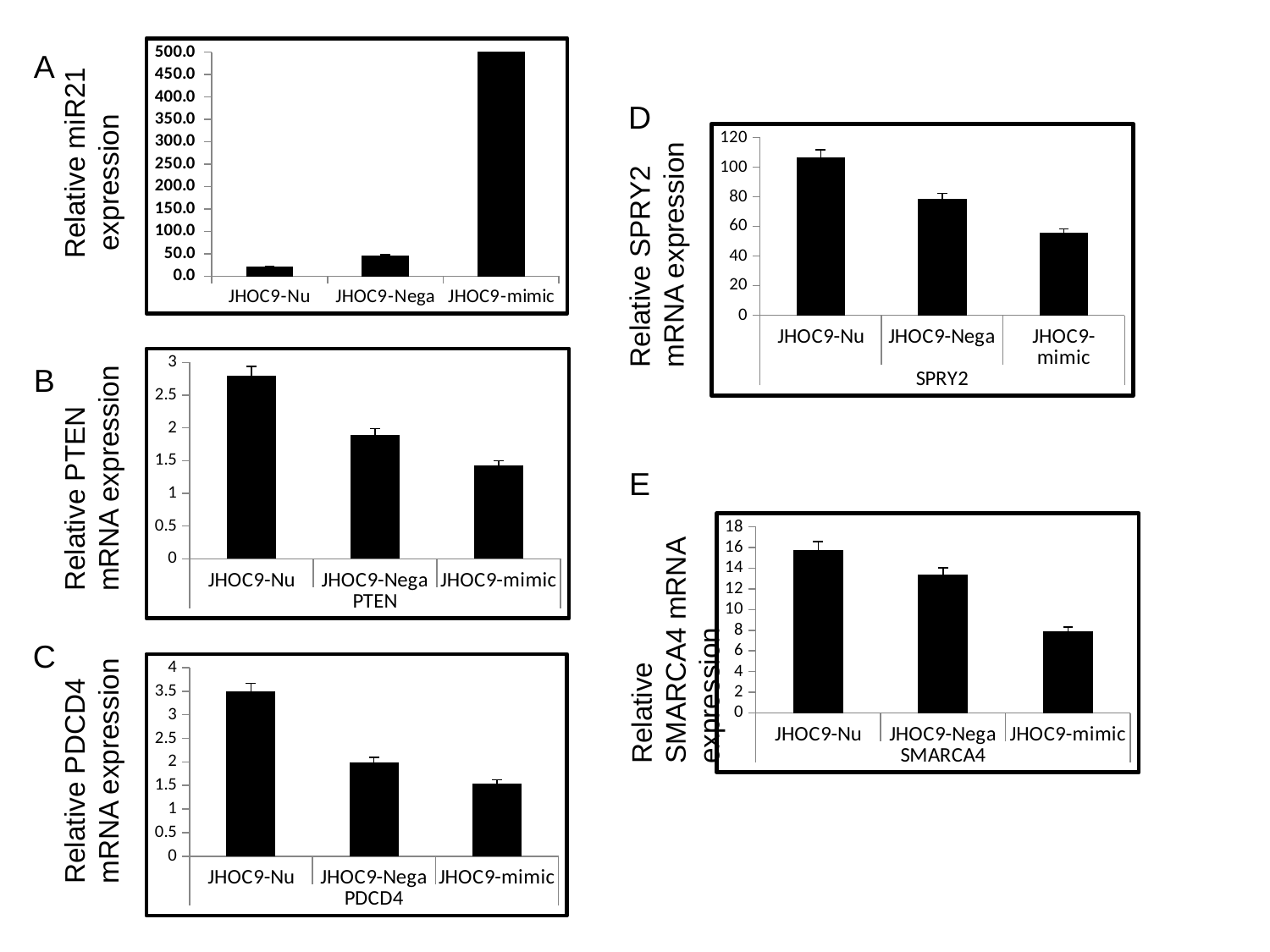

### Chart
| Category | |
|---|---|
| JHOC9-Nu | 20.224093852885677 |
| JHOC9-Nega | 45.166401717089336 |
| JHOC9-mimic | 15640.382410487131 |A
Relative miR21
 expression
D
### Chart
| Category | |
|---|---|
| JHOC9-Nu | 106.39765149746384 |
| JHOC9-Nega | 78.41526167802719 |
| JHOC9-mimic | 55.62414098166303 |Relative SPRY2
mRNA expression
### Chart
| Category | |
|---|---|
| JHOC9-Nu | 2.7974067938218217 |
| JHOC9-Nega | 1.8973485602875364 |
| JHOC9-mimic | 1.42852 |B
Relative PTEN
mRNA expression
E
Relative SMARCA4 mRNA expression
### Chart
| Category | |
|---|---|
| JHOC9-Nu | 15.792169641393492 |
| JHOC9-Nega | 13.360276055761194 |
| JHOC9-mimic | 7.931432552231688 |C
### Chart
| Category | |
|---|---|
| JHOC9-Nu | 3.4925123907999973 |
| JHOC9-Nega | 1.9941468021939281 |
| JHOC9-mimic | 1.54684546 |Relative PDCD4
mRNA expression
